# Rational Design, Synthesis, and Evaluation of Fluorescent CB_2_ Receptor Ligands for Live-Cell Imaging: A Comprehensive Review

**DOI:** 10.3390/ph16091235

**Published:** 2023-08-31

**Authors:** Pinaki Bhattacharjee, Malliga R. Iyer

**Affiliations:** Section on Medicinal Chemistry, National Institute on Alcohol Abuse and Alcoholism, National Institutes of Health, 5625 Fishers Lane, Rockville, MD 20852, USA

**Keywords:** endocannabinoid system, cannabinoid type 2 receptor, fluorescent probe, disease

## Abstract

The cannabinoid receptors CB_1_ and CB_2_ are class A G protein-coupled receptors (GPCRs) that are activated via endogenous lipids called endocannabinoids. The endocannabinoid system (ECS) plays a critical role in the regulation of several physiological states and a wide range of diseases. In recent years, drug discovery approaches targeting the cannabinoid type 2 receptor (CB_2_R) have gained prominence. Particular attention has been given to selective agonists targeting the CB_2_ receptors to circumvent the neuropsychotropic side effects associated with CB_1_ receptors. The pharmacological modulation of CB_2_R holds therapeutic promise for various diseases, such as inflammatory disorders and immunological conditions, as well as pain management and cancer treatment. Recently, the utilization of fluorescent probes has emerged as a valuable technique for investigating the interactions between ligands and proteins at an exceptional level of spatial and temporal precision. In this review, we aim to examine the progress made in the development of fluorescent probes targeting CB_2_ receptors and highlight their significance in facilitating the successful clinical translation of CB_2_R-based therapies.

## 1. Introduction

The G protein-coupled receptors (GPCRs) are a class of over 700 proteins that include seven transmembrane domains and are produced on the cellular membrane [1]. These cellular components are responsible for transmitting extracellular signals originating from many stimuli, including light, peptide hormones, and neurotransmitters [2,3]. This transmission enables intracellular second messenger systems to activate, facilitating cellular responses to the surrounding environment. G protein-coupled receptors (GPCRs) play a crucial role in the control of several physiological processes, including vision, behavior, mood, energy balance, immunology, and inflammation [4,5,6].

In recent years, research into the endocannabinoid system (ECS) has expanded significantly among the scientific community. The endocannabinoid system has been investigated for its potential role in a variety of pathophysiological disorders due to its wide range of regulatory effects [7,8]. The cannabinoid receptors, their endogenous lipid ligands (endocannabinoids), and the machinery for their manufacture and metabolism are all parts of the endocannabinoid system. The cannabinoid receptors CB_1_ and CB_2_ have been extensively studied and recognized as prominent members of the cannabinoid receptor family. These receptors are classified as G protein-coupled receptors (GPCRs), and their primary function involves coupling with inhibitory G proteins. The primary interaction of CB_1_ and CB_2_ receptors is coupling to inhibitory G proteins (G_i_/G_o_) and activation of the signaling pathways associated with G_i_/G_o_ [9]. These receptors are activated via both exogenous and endogenous ligands. Apart from being the molecular targets of the exogenous psychoactive component of Cannabis sativa, ∆^9^-tetrahydrocannabinol (THC), the endocannabinoid system is also influenced by naturally occurring endocannabinoids, such as anandamide (AEA) and 2-arachindonyl glycerol (AEA). The cannabinoid receptors CB_1_ and CB_2_ play an important role in the physiological control of numerous central and peripheral activities [10]. The CB_1_ receptor comprises a total of 472 amino acids, is highly expressed in the central nervous system, and is particularly abundant in various brain regions, including the hippocampus, cerebellum, cortex, and basal ganglia [11]. Additionally, CB_1_ receptors are found in peripheral tissues such as the liver, lung, pancreas, and ileum [12].

The CB_2_ protein encodes the CNR2 gene (GeneID1269) located in 1p36.11.; it consists of a total of 360 amino acids [13]. CB_2_R has been increasingly reported in brain pathologies, but its highest concentration is found in immune system cells [14,15]. These receptors play a crucial role in the immunomodulatory effects induced via cannabinoids. The two cannabinoid receptors exhibited a 44% level of homology and a 68% degree of sequence similarity inside their transmembrane regions [16].

There are significant variations across species, as demonstrated by the approximately 80% similarity in amino acid sequences between humans and rats. Two isoforms of CB_2_ have been discovered in humans, namely CB_2_A and CB_2_B. CB_2_A is primarily found in the testis and is also expressed, albeit to a lesser extent, in certain regions of the brain, such as the amygdala, caudate, putamen, nucleus accumbens, cortex, hippocampus, and cerebellum. On the other hand, CB_2_B is the main isoform found in the spleen and leukocytes, but it is also present in other peripheral tissues [13,17].

Although CB_1_R and CB_2_R have enormous therapeutic promise, very few medicines that target them are currently available on the market. The similarities between the active-state orthosteric ligand-binding pockets of CB_1_R and CB_2_R make it challenging to develop selective ligands based on their activation [18,19,20]. Despite their importance, very little is known about the signaling processes involved or the patterns of expression in different tissues. Therefore, a comprehensive knowledge of CB_1_R and CB_2_R tissue expressions and their signaling pathways is critical to avoid off-target or undesired psychotropic effects. Hence, subtype-selective probes are the most effective means of addressing these problems. CB_2_R agonists have shown vast potential, offering directions for future selective medication development [17,21,22]. Understanding ligand–protein interactions, target engagement, and the mechanism of action is crucial to the development of novel medications. GPCR expression in endogenous tissue under physiological conditions is limited, making it challenging to determine target interactions. The adaptability of proteins when activated emphasizes this even further. Biomarkers are a valuable tool for determining target engagement in vivo. Although it has been hypothesized that alterations in the ECS might be used as a biomarker for psychiatry, there are presently no biomarkers for CB_2_R, which explains the necessity of specialized imaging techniques with minimal off-target interactions [23]. The imaging of CB_2_R in live cells remains underexplored due to its limited expression in healthy tissue and the lack of robust pharmacological techniques [24].

Fluorescent compounds have allowed for significant advances in the visualization of proteins, receptors, and ion channels within and outside of cells. Using these molecules, researchers have gained insight into the subcellular localization and, in certain cases, dynamic mobility of a number of membrane-bound proteins. Typically, antibodies or the overexpression of a fluorescent protein linked to a target protein are employed to mark membrane-bound proteins [25].

Molecular probes that have been fluorescently tagged have found widespread use in both in vitro and in vivo settings. Finding an area where chemical modifications may be made without affecting probe affinity is the primary difficulty in designing fluorescent probes. Traditional antibodies are unable to see intracellular protein targets, but small-molecule-derived probes are capable due to the incorporation of cell-permeable fluorescent dyes. By using them, we may learn about the molecular processes behind pharmacological responses alongside where and how frequently targets are expressed. To better understand how ligands interact with their targets, methods such as fluorescent confocal microscopy, flow cytometry, and time-resolved Förster resonance energy transfer (TR-FRET) have been employed [24].

Recent efforts to investigate CB_2_R expression at the tissue level have relied on positron emission tomography (PET) tracers; however, this approach lacks the necessary cellular resolution. PET probes need a reduced nanomolar affinity in order to be suitable for imaging applications, sometimes leading to false-positive outcomes [26,27] Fluorescent imaging probes, on the other hand, have recently emerged as powerful instruments for studying the subcellular localization, structure, dynamics, and function of proteins and GPCRs in live cells. Such probes also have the potential to provide high-throughput data on binding kinetics and equilibrium without the use of radioactive materials. Fluorescence is often used in the investigation of small molecule–target interactions, facilitated via techniques such as fluorescence resonance energy transfer (FRET) and bioluminescence resonance energy transfer (BRET), which are widely utilized in this field. Both the bioluminescence resonance energy transfer (BRET) and fluorescence resonance energy transfer (FRET) techniques have been used in the viewing of various G protein-coupled receptors (GPCRs), therefore illustrating the suitability of both approaches for ligand–protein imaging. As a result, the precise evaluation of CB_2_R pharmacology in live cells is still hampered by the limited robust antibodies, the reversible high-affinity probe with desirable fluorescence features, and the requisite specificity for CB_2_R. Phytocannabinoid-derived CB_2_R ligands, which are typically used as starting points for probe development, present significant challenges due to their highly lipophilic nature. When combined with the usage of highly lipophilic dyes, this may have a synergistic effect that reduces the dyes’ overall characteristics and makes them less useful for a wide range of applications. Furthermore, the fluorescent probe should not have any interspecies variations between rodent and human CB_2_Rs to provide excellent translatability of preclinical pharmacological animal data to the clinic and perhaps deploy a CB_2_R fluorescence probe for dosage selection in humans [27,28,29,30,31].

## 2. CB_2_ Receptor: Molecular Characterization and Spatial Distribution

Cannabinoids have an influence on almost all physiological systems in the human body. Unsurprisingly, extensive research has shown the presence of CB receptors throughout the human body. The first categorization assigned reactions inside the central nervous system (CNS) to the CB_1_ receptor, whereas those occurring in the peripheral regions were attributed to the CB_2_ receptor. As previously mentioned, the CB_2_ receptor was first cloned from a human leukocyte cell line, and it has been shown that the CB_2_ receptor is expressed in peripheral blood leukocytes to variable degrees [10,32].

**Figure 1:**
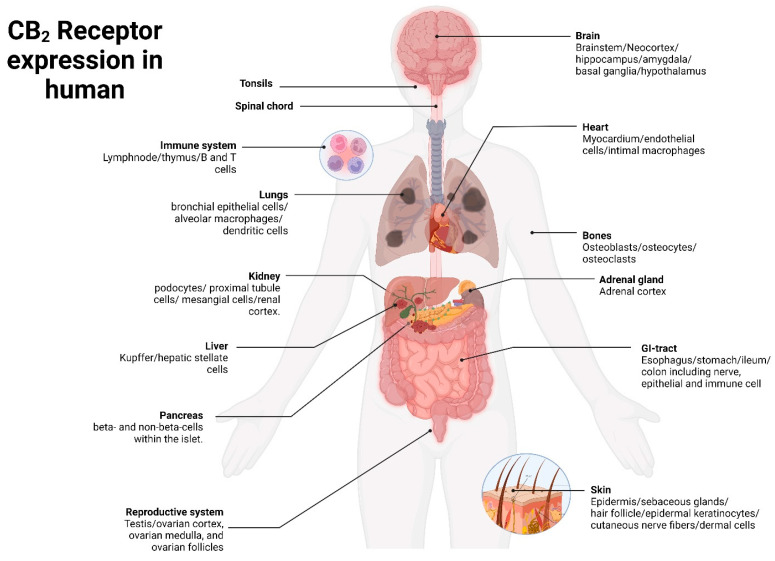
CB_2_ receptor expression in human physiological system.

The most prominent manifestation is seen on B-cells, with natural killer (NK) cells exhibiting a similar level of expression in close proximity. Myeloid cells have moderate levels of receptor expression, whereas resting T cell subsets show low levels of receptor expression. It is not unexpected that certain components of the immune system, including the tonsils, spleen, thymus, and lymph nodes, have significantly elevated levels of CB_2_ receptor mRNA expression. A comprehensive investigation has provided more evidence that the CB_2_ receptor is present in distinct areas of the brain, spinal cord, and dorsal root ganglia, as well as the gastrointestinal (GI) tract, liver, bone, and reproductive systems (Figure 1) [33,34].

The comprehensive understanding of the CB_2_ receptor’s existence and function inside the central nervous system remains inadequate. Initially, there was a prevailing belief that the expression of CB_2_ receptors was absent in nonimmune cells of the central nervous system. This belief stemmed from the findings of Munro et al., who, during their cloning of the receptor, failed to locate CB_2_ receptor mRNA in any region of the brain [10]. However, many studies have recently shown that CB_2_R plays a functionally relevant role in the central nervous system and various pathologies associated with the brain [34,35,36,37].

**Figure 2:**
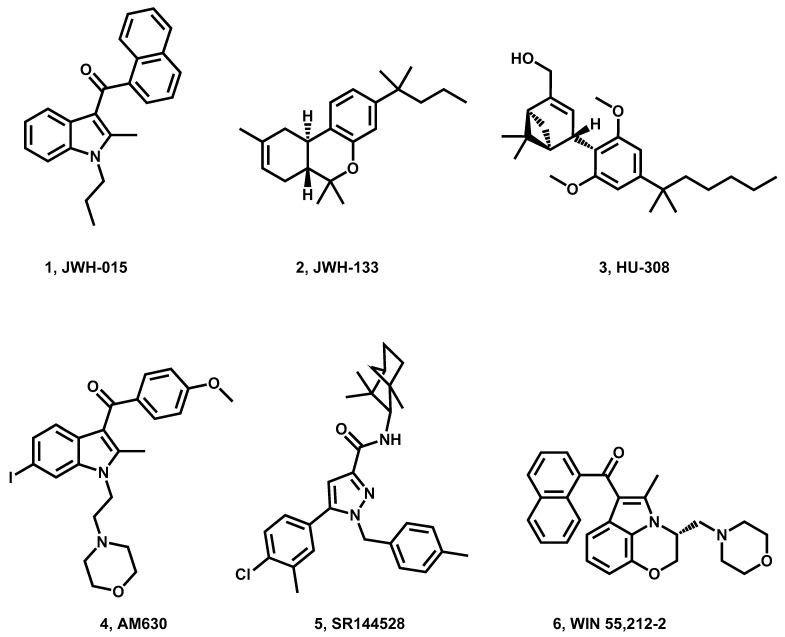
Examples of CB_2_R probe (agonist/antagonist).

The CB_2_ receptors located in peripheral tissues and cells, including those of the immune system, hematopoietic cells, bone, liver, peripheral nerve terminals, and keratinocytes, play a crucial role in the suppression of cytokine/chemokine secretion, as well as the migration of neutrophils and macrophages. Additionally, they contribute to the deceleration of persistent inflammatory mechanisms and the regulation of chronic pain [38]. The limited dependability of the antibodies used in immunochemistry investigations to study CB_2_R receptor expression underscores the need for further investigation to enhance our understanding of the participation of the CB_2_ receptor in the central nervous system and neuroinflammation. Numerous CB_2_ ligands have been synthesized, and their receptor function has been studied in detail (Figure 2); however, drugs solely targeting CB_2_ have not yet made it to the clinic [16,17,22].

The therapeutic potential of CB_2_ and its role in various pathologies have been extensively reviewed [17,21,39,40,41,42]. Our focus here is to review the imaging probes that were developed to study CB_2_ expression and function, as well as to highlight challenges and opportunities to guide future improvements.

## 3. Fluorescent Probe Design Principles

Numerous CB_2_ agonists have been documented in the scientific literature, and an in-depth investigation of patents revealed the existence of several CB_2_ receptor modulators now undergoing clinical development at varying stages [22,43]. To study the brain CB_2_ receptors effectively, the ligand must demonstrate the capability to effectively penetrate the blood–brain barrier, enabling it to exercise its intended effects on activated microglia [28]. Furthermore, for its effective clinical translation, it must undergo rigorous testing with the right patient cohort [42].

The CB_2_ receptor has garnered significant interest as a promising candidate for the noninvasive detection of neuroinflammation due to its reported upregulation in resident microglial cells after cerebral ischemia, injury, and other neuroinflammatory disorders. When creating small-molecule fluorescent ligands, it is essential to consider many parameters. The successful interaction between the ligand and the fluorophore is crucial for both specific binding to the desired target and the generation of a strong fluorescence signal, enabling effective imaging of the receptor. The pharmacophore must possess high potency and selectivity while also accommodating chemical functionalization by coupling to a linker and fluorophore. Functionalization may be included in the system via chemical methods in certain cases. The determination of spacer length should ideally be derived from an optimization procedure. However, limited research is available that has provided a systematic review of the length of the spacer used for the fluorescent probe being investigated [44,45].

Based on an extensive review of the existing literature, it is evident that spacers are composed of a minimum of six atoms [46]. Extended linkers have been used to accommodate larger fluorophores, such as near-infrared fluorophores (NIRF) or quantum dots (QDs) [47]. The physicochemical parameters of the fluorescent probe may be influenced by the chemical composition of the linker. Multiple investigations have posited that the presence of elevated lipophilicity could play a role in the nonspecific binding of some fluorescent probes [48,49].

The best selection of a fluorophore should prioritize characteristics such as high fluorescence intensity, photostability, and compatibility with the available microscope and the specific system under investigation. Commonly used fluorophores for fluorescence imaging include fluorescein, coumarin, dansyl, cyanine, rhodamine derivatives, and NBD, as previously indicated. When selecting an appropriate fluorophore, it is crucial to ensure that the excitation and emission wavelengths do not fall within the spectral range where cells demonstrate significant amounts of autofluorescence and light scattering. It is essential to exercise caution in order to prevent interference from pre-existing fluorescent signals inside the target cells, including those associated with a GFP-tagged protein [50]. The fluorescent ligand’s modular design approach, as previously described by Gazzi et al., encompassed three primary components: (i) a recognition element (pharmacophore) capable of accommodating subsequent chemical functionalization while retaining its affinity and selectivity towards the target; (ii) a suitable fluorescent dye; and (iii) a linker that serves to separate these two functionalities. This enables the positioning of the dye in the extracellular area, external to the receptor. The incorrect escape vector provided via the linker attachment, when combined with a big and charged fluorescent dye that is typically larger than the recognition element, results in a significant disruption of the interaction between the receptor and the ligand and may even cause a total loss of binding affinity. Hence, the careful identification of optimal linker attachment sites, together with the subsequent optimization of linker length and composition, is of utmost importance in order to prevent any adverse interactions with the receptor [25,51,52].

## 4. CB_2_R Targeting Fluorescent Probes

Even though CB_2_R expression is significantly increased in pathological conditions, no particular drugs targeting receptors have been introduced to the market despite its obvious relevance. This knowledge gap is largely due to a lack of understanding of receptor biology, particularly in the context of tissue and disease. The problem is made worse by very low expression levels in native cells, a dearth of authorized chemical probes, and a paucity of tailored antibodies [53,54,55,56].

Given the aforementioned problems, researchers have been diligently endeavoring to produce chemical tools that may address the unresolved questions in CB_2_R pharmacology. This effort aims to optimize and facilitate the creation of effective medications targeting CB_2_R. Previous literature exists in which authors have documented advancements in the development of selective CB_2_R ligands that have been refined for use as pharmacological and imaging instruments, such as positron emission tomography (PET), fluorescent, photochromic, and covalent chemical probes [27].

These probes are expected to enhance our perception of the receptor’s activities and its role in regulating the physiological processes and pathophysiological pathways associated with various diseases. This, in turn, will contribute to the advancement of our imaging techniques and knowledge of the receptor’s behavior. The focus of research has been on enhancing the progress of developing specific fluorescent ligands for CB_2_R. This is because the current techniques used to study CB_2_R, such as radioligands and antibodies, have certain limitations [57].

Fluorescent ligands are powerful tools for visualizing receptor dynamics in living cells, and they are compatible with methods like confocal microscopy, fluorescence resonance energy transfer, and flow cytometry [29].

The use of the identified CB_2_R fluorescent ligands as imaging tools has been impeded in the past due to the presence of significant nonspecific binding at membranes. The lipophilicity of cannabinoids and their pharmacophore makes it challenging to develop fluorescent ligands that are devoid of nonspecific binding. Despite these challenges, researchers aim to strike an equilibrium between pharmacological selectivity and physicochemical properties through linker modifications connecting the pharmacophore with fluorescent dyes [58].

### 4.1. Fluorescent Indole Derivatives

**Figure 3:**
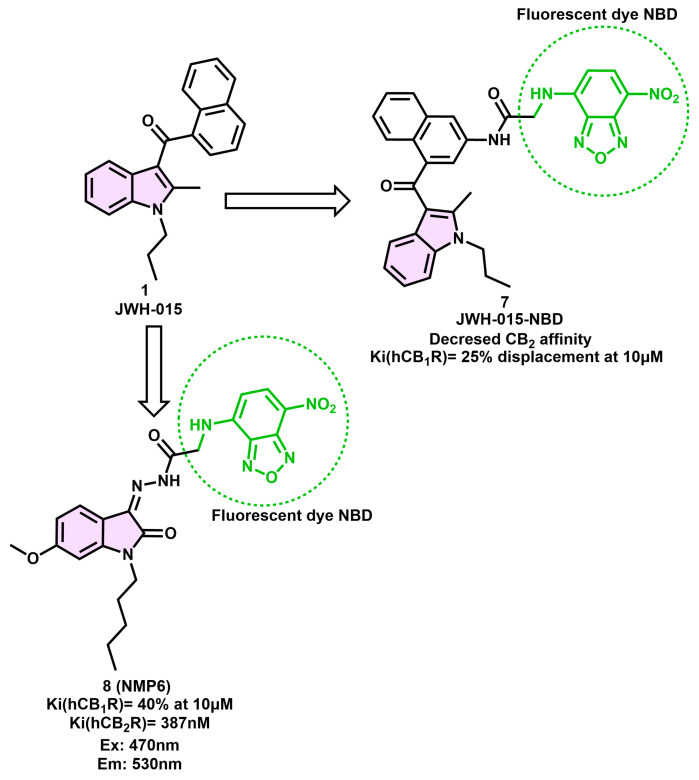
Indole-based CB_2_R fluorescent probe.

Indoles have been extensively used as fluorescent ligands among the CB_2_ receptor pharmacophores. This is mostly due to the presence of the indole moiety in many CB_2_R reference ligands, including JWH-015 (**1**) and WIN 55, 212-2 (**6**). JWH-015 is a very potent agonist of the cannabinoid receptor characterized by the presence of a naphthoylindole scaffold (Figure 3). It has about 28 times more of an affinity for the CB_2_ receptor compared to the CB_1_ receptor. The first development of fluorophores for cellular imaging was the invention and synthesis of a molecule that incorporated the fluorescent dye nitrobenzofurazan (NBD), owing to its notable selectivity. The NBD fluorophore was conjugated to the naphthalene moiety via a brief amide linkage in order to inhibit the formation of a nonfluorescent molecule with PET activity. The affinity of CB_2_R of **7** was assessed using a displacement test against [^3^H]-CP 55,940 in CHO cells that were engineered to express CB_2_R. The novel compound NBD JWH-015 (**7**) exhibited a displacement rate of just 25% at a concentration of 10 μM. Unfortunately, the generated probe exhibited a significant decrease in CB_2_R affinity, exceeding a 250-fold reduction compared to the untagged ligand. Consequently, its suitability as a selective CB_2_R fluorescence instrument was compromised. Upon visualization using a confocal microscope, it was seen that the majority of the fluorescence was concentrated inside the cytoplasm. This localization may be attributed to the compound’s lipophilic nature, which facilitated its quick absorption via the cells. The compound’s precursors were discovered to retain CB_2_R activity; however, this aspect was not further investigated. Instead, subsequent studies have shown that indole compounds mostly exhibit tolerance for fluorophores at position C-7 despite their antagonistic properties [59].

The reduced affinity prompted the use of a powerful CB_2_R antagonist scaffold, namely the 6-methoxy-N-pentyl isatin acylhydrazone, which was then linked with the NBD dye (Figure 3) [60]. The synthesis of NMP6 (**8**) was carried out through a two-step process, wherein NBD was used to bind to the sizable hydrophobic pocket, hence potentially enhancing CB_2_R affinity. In the competitive binding experiment conducted on Chinese hamster ovary (CHO-K1) cells, NMP6 (**8**) exhibited a Ki value of 387 nM when tested against [^3^H]-CP 55,940. The excitation and emission wavelengths of NMP6 (**8**) in acetonitrile were measured to be 470 nm and 530 nm, respectively. NMP6 continued to exhibit nanomolar affinity and good selectivity for CB_2_R, therefore prompting further evaluation in CB_2_R cellular imaging experiments. NMP6 (**8**) demonstrated its ability to detect CB_2_R via the use of confocal microscopy in nontransfected cells and cytometric analysis in B lymphocytes. The binding of CB_2_R was seen to be impeded in competition experiments or when preincubated with GW842166X, a selective CB_2_R agonist. These findings provide evidence of the selectivity of NMP6 (**8**) towards CB_2_R. Although the scaffold has shown a consistent affinity for CB_2_R, there has been a lack of exploration about the use of other analogs incorporating the isatin scaffold for CB imaging [61].

The functionalization of the other locations on the indole ring was also examined in order to produce fluorescent ligands. The results of N1 functionalization experiments indicate that linkers longer than a hexamethylene chain are not well tolerated. However, further investigation into the C5-7 positions using various linkers has yielded promising compounds. Notably, a consistent pattern has emerged: C5-substituted indoles exhibit agonistic activity, while the C7-substituted derivatives demonstrate inverse agonistic activity as observed in a cAMP assay. The use of positions 5 and 7 of the indole moieties was employed for the purpose of the prospective attachment of linkers and fluorophores. Following a structure–activity relationship (SAR) investigation of the indole scaffold, Cooper et al. discovered ligands for the CB_2_R receptor that exhibited a nanomolar affinity [61]. Notably, these ligands had bulky substituents at either position 1 or 3 of the indole scaffold, such as ethyl morpholine, methoxyphenyl, or methyl tetrahydropyran. However, the binding between CB_2_R and the linkers was significantly impeded when decorated with BODIPY 630/650-X fluorescent tags in both places. Regrettably, the conjugation of both these indole derivatives with the fluorophore BODIPY 630/650-X in either the C5 or C7 positions via alkylene, PEG, or dipeptide linkers, respectively, yielded fluorescent ligands lacking an affinity for CB_2_R (Ki > 10,000 nM). This outcome indicates that only smaller substituents are permissible at these specific locations. In light of the commitments made via the C5 or C7 functionalized indole derivatives, it is possible to explore the same sites using fluorescent tags other than BODIPY.

### 4.2. Pyrazole-Based Fluorescent Probes

The subclass of fluorescent CB_2_R ligands is derived from various fluorophore enhancements included in the biarylpyrazole framework of SR 144528 (**5**). In 1998, Rinaldi-Carmona et al. developed the first selective CB_2_R antagonist with a high affinity (Ki CB_2_R = 0.6 nM), which was later shown to act as an inverse agonist [62].

Nevertheless, although it possesses a pharmacological profile that is very desirable, it is deficient in functional groups that would facilitate convenient fluorophore conjugation. In the year 2008, the compound MBC94 (**9**) was first produced, whereby a benzyl bromide group was substituted for a methyl group to enhance its coupling capabilities. The compound has a terminal amino group that may be readily conjugated to imaging scaffolds, resulting in a modest decrease in affinity by a factor of 15 (K_i_ = 15 nM) [63,64].

The coupling of the near-infrared (NIR) dye IRDye 800CW NHS ester to MBC94 (**9**) resulted in the formation of NIRmbc94 (**10**). This compound represents the first instance of a CB_2_R selective probe and is among the earliest fluorescent ligands for CB_2_R, exhibiting a high affinity in the nanomolar range (K_i_ = 260 nM). The probe has favorable photophysical characteristics, demonstrating a peak absorbance at 779 nm and emitting light at 797 nm when placed in a water medium. The NIR microscopy studies exhibited favorable imaging capabilities, as shown by a signal-to-noise ratio (S/N) of 1.6. Moreover, it demonstrated an intriguing pharmacological profile due to its absence of nonspecific binding and its excellent selectivity in competitive binding studies with SR144528 as the displacing ligand.

CB_2_-expressing DBT cells derived from a mouse astrocytoma cell line were labeled with NIR-mbc94 (**10**), but no signal was seen when the NIR dye was incubated in the cells. The fluorescence signal exhibited a decrease in intensity when cells lacking CB_2_R expression were subjected to incubation with an equivalent dose. All the experimental results provided support for the possible use of NIR-mbc94 (**10**) in conducting the high-throughput screening (HTS) of chemical libraries including compounds that bind to CB_2_R. An initial evaluation of chemicals was conducted, confirming the validity of this theory and indicating potential new discoveries. Nevertheless, there are several drawbacks associated with NIR-mbc94 (**10**), mostly stemming from the use of its fluorophore, IRDye 800CW. This particular dye is known for its high cost and notable stability concerns. Zhang et al. successfully addressed these challenges by selecting NIR760 as a novel probe for conjugation with MBC-94 (**9**) [65].

**Figure 4:**
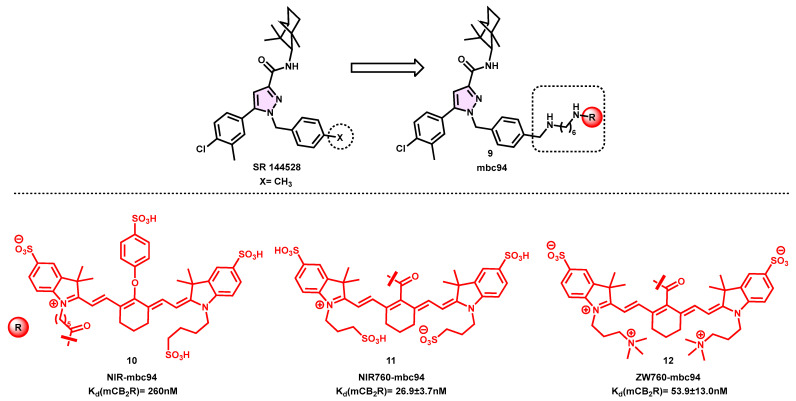
Biarylpyrazole-based CB_2_ fluorescent probe.

The recently developed fluorophore, NIR760, has comparable characteristics to IR Dye 800CW, although with a different linker location relative to the pharmacophore. The absorbance of the novel fluorescent probe is measured at a wavelength of 766 nm, while its fluorescence emission occurs at 785 nm, exhibiting a quantum yield of 15.2%. The recently developed compound NIR760-mbc94 (**11**) exhibited a high level of affinity in the nanomolar range, as seen in its dissociation constant (K_d_) of 26.9 nM. Additionally, it demonstrated a considerable degree of specific binding, as indicated by a 40% drop in fluorescence intensity following a 30-min pretreatment with 10 µM of SR144528, as depicted in Figure 4. Significantly, the CB_2_R fluorescent probe NIR760-mbc94 (**11**) was used as the first agent for in vivo testing in a CB_2_-mid DBT murine tumor model with the aim of evaluating its viability as a tool for cancer imaging. The maximal absorption of the tumor was seen 24 h after intravenous injection, but NIR760-mbc94 (**11**) exhibited the highest ratio of the tumor area to the normal area (T/N) after 72 h, progressively rising over time. This finding provides evidence of a prolonged half-life. The fluorescent CB_2_R probe NIR760-mbc94 (**11**) was subjected to testing for inflammatory imaging both in vitro and in vivo. The novel fluorescent ligand was used in the in vivo tests to investigate an inflammatory mouse model generated via a complete Freund’s adjuvant (CFA). An evaluation of specific binding at the cellular level was conducted on ordinary RAW-264.7 (mouse macrophages) and LPS-activated cell lines. The antagonist SR144528 was used, resulting in decreases in the fluorescence signal of 36% in ordinary cells and 23% in activated cells. The use of in vivo imaging in a mouse inflammation model revealed a progressive enhancement in image contrast over time, specifically in the fluorescence identified in an inflamed paw compared to an uninflamed paw. This finding serves to validate the previously observed metabolic stability and indicates a peak blockage of 30% occurring at the 36-h mark. The use of immunofluorescence labeling on frozen slices of paw tissue confirmed prior findings while also reiterating the modest level of specific binding shown by NIRmbc94 (**10**) [66].

The authors propose that the net negative charge of the NIR760 probe may be responsible for certain limitations. To address this issue, they have made advancements by creating a zwitterionic CB_2_R NIR probe named ZW760-mbc94 (**12**). This new probe retains a similar structure to NIR760 but substitutes alkyl sulfonic acid groups with trimethyl amine cations. The compound ZW760-mbc94 has a strong binding affinity to the CB_2_R receptor with a measured dissociation constant (Kd) of 53.9 nanomolar (nM). As anticipated, the introduction of ZW760-mbc94 (**12**) to CB_2_-mid DBT cells was hindered by about 50% when 4-quinolone-3-carboxamide (4Q3C), a specific CB_2_R blocking agent, was used. This inhibition was greater than the earlier outcome seen with NIR760-mbc94. In an experimental study using an in vivo mouse model, CB_2_-mid DBT tumor cells were injected subcutaneously. The fluorescence intensity of ZW760-mbc94 (**12**) was shown to be greater in comparison to animals that were pre-injected with 4Q3C to block certain receptors. Notably, ZW760-mbc94 (**12**) exhibited significant absorption in the liver and kidneys, which are organs involved in metabolism. In the ex vivo biodistribution research, a discernible inhibitory impact of around 47% was only found in the cancer cells. This finding suggests that ZW760-mbc94 (**12**), in comparison to its precursor chemical NIR760-mbc94 (**11**), has enhanced and targeted binding capabilities at both the cellular and animal levels [67].

**Figure 5:**
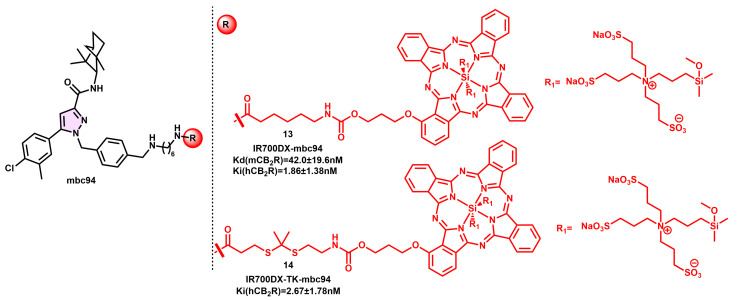
MBC94 CB_2_R-selective fluorescent derivatives with various NIR fluorophores.

The previously mentioned research team successfully developed a novel phototherapeutic alternative, known as IR700DX-mbc94 (**13**), using the near-infrared (NIR) probe IR700DX. This innovative approach shows promise in targeting tumor cells that exhibit high levels of CB_2_R expression (Figure 5) [68].

The compound exhibited absorbance and emission wavelengths of 682 and 690 nm, respectively, when dissolved in methanol. The nontoxicity of IR700DX-mbc94 (**13**) was established by the absence of irradiation in both CHO-K1/CB_2_ and CB_2_-mid DBT cells. The chemical had a dissociation constant (K_d_) of 42.0 ± 19.6 nM and indicated cytotoxic and inhibitory effects on CB_2_ positive cells upon irradiation, contingent upon its binding to CB_2_R. Research was conducted to investigate targeted phototherapy in vivo utilizing IR700DX-mbc94 (**13**). The results of this investigation demonstrated the production of singlet oxygen (^1^O_2_) and free radicals under irradiation, leading to a kind of cell death resembling necrosis [69].

The IR700DX-mbc94 (**13**) compound has promising characteristics as a possible agent for selective phototherapy in cancer cells. It demonstrates little cytotoxicity prior to irradiation and exhibits a preference for CB_2_ + cells. The IR700DX-mbc94 (**13**) compound was subsequently altered to include a dimethyl thioketal moiety inside the linker region. The recently discovered probe IR700DX-TK-mbc94 (**14**) exhibited a high affinity in the nanomolar range for displacing [^3^H]-CP55,940 from hCB_2_R, similar to the affinity seen with IR700DX-mbc94 (**13**) (Figure 5). In the CB_2_R+ animal model of a delayed brain tumor, CB_2_-mid DBT cells treated with IR700DX-TK-mbc94 (**14**) showed a significant 59% reduction in cell viability after irradiation. In contrast, IR700DX-mbc94 (**13**) treatment resulted in little cell death. The observed increase in cell mortality when exposed to IR700DX-TK-mbc94 (**14**) was constant across all incubation durations and concentrations.

The analysis of structure–activity relationships (SARs) revealed that the chromenopyrazole scaffold exhibits tolerance towards N-pyrazole aromatic and alkyl substituents. However, this tolerance leads to the development of high-affinity ligands that are selective for CB_1_R, selective for CB_2_R, or nonselective [31,69]. The study documented the enhancement of CB_2_R selectivity via the phenolic alkylation of chromenopyrazole. Nevertheless, the presence of both the N-phenyl and O-alkyl groups in the same ligand resulted in a reduction in CBR affinity.

**Figure 6:**
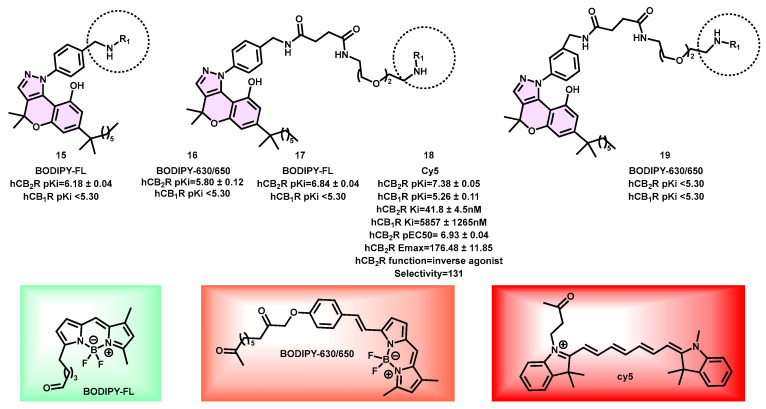
Pyrazoline-based CB_2_ fluorescent probe.

Several CB_2_ fluorescent probes were produced, and wide-field fluorescence microscopy imaging tests were conducted to assess the capability of probe **18** in identifying CB_2_R at the individual cell level. The fluorescent ligand with the greatest affinity was Cy5-containing (**18**) (hCB_2_R pK_i_ = 7.38 ± 0.05, Ki = 41.8 ± 4.5 nM at hCB_2_R; 5857 ± 1265 nM at hCB_1_R), exhibiting a selectivity of 131-fold over CB_1_R (Figure 6). In the context of a cAMP BRET experiment, compound **18** exhibited strong potency as an inverse agonist for the CB_2_R receptor [31]. The HEK-293 cells expressing CB_2_R were subjected to incubation with a concentration of 1 μM of the fluorescent ligand **18**. This resulted in evident labeling on the cell surface, but no noticeable accumulation was seen inside the cells. The specific binding of compound **18** to the CB_2_R receptor was shown by the little fluorescence detected when the cells were co-incubated with a high affinity, nonfluorescent CB_2_R inverse agonist, SR144528 (30 μM), and compound **18**. The specific binding of CB_2_R was further confirmed by the little fluorescence produced when incubating **18** with HEK-293 cells that were transfected with an empty vector, indicating the absence of CB_2_R. Given that **18** functions as an inverse agonist, the probability of receptor internalization is deemed exceedingly improbable. Moreover, considering the physical attributes of **18**, such as its substantial molecular weight and extensive polar surface area, the ease of cellular permeability seems implausible. In the imaging experiments, surface CB_2_R-restricted labeling was observed, which was confirmed by the presence of clear colocalization with cell surface CB_2_R. This colocalization was detected using a non-cell-permeable primary antibody that specifically targeted an extracellular epitope tag on the receptor. Furthermore, it became apparent that there was an absence of intracellular labeling despite the established knowledge that CB2R is expressed intracellularly in the absence of ligand activation. Fluorescent ligand **18** has a high affinity for CB_2_R, demonstrating selectivity and powerful inverse agonist action. Additionally, it possesses excellent imaging capabilities. Consequently, it is anticipated that this ligand will prove to be a significant tool for in vitro and ex vivo investigations, particularly in the context of examining CB_2_R expression in whole-cell binding applications.

### 4.3. Fluorescent Oxoquinoline Derivatives

The use of oxoquinolines has been extensively employed in the acquisition of highly specific and powerful CB_2_R ligands [70].

Consequently, a substantial number of comprehensive SARs have been documented for this particular subclass, enabling the development of fluorescence instruments based on oxoquinoline with notable affinity and selectivity. In the first instances of success, the fluorophore, namely NIR760, was inserted directly into the aromatic moiety using an appropriate linker. The pyrazolo [1,5-a]pyrimidin-7-one moiety has been shown to have a strong affinity for CB_2_R binding and selectivity while also possessing the advantageous characteristic of being highly tolerant to chemical modifications at the C2 position [71].

**Figure 7:**
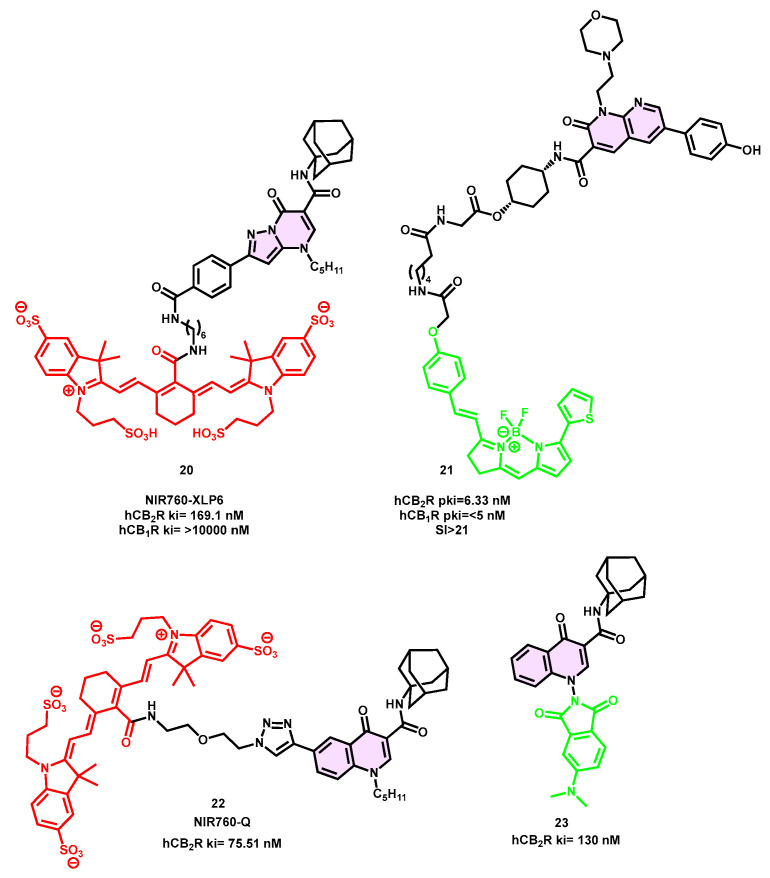
Oxoquinoline-based fluorescent probe for CB_2_R.

NIR760-XLP6 (**20**) was synthesized by attaching NIR760 to the pyrazolo [1,5-a]pyrimidin-7-one core using a 4 aminohexylphenylamide linker (Figure 7) [72]. The study demonstrated the significant affinity and selectivity of CB_2_R (with a dissociation constant, Kd, of 169.1 nM) in the relevant DBT-CBR cells. This was supported by the presence of nonspecific binding, which was validated using in vitro fluorescence imaging. Both the binding test and biodistribution investigation yielded further data supporting the favorable and specific binding of NIR760-XLP6 (**20**) to CB_2_R. Significantly, the measured tumor-to-normal (T/N) ratio reached a maximum value of 7.9 in the DBT-CB_2_ tumor at 72 h after intravenous administration. The preference of NIR760-XLP6 (**20**) for CB_2_R over CB_1_R in a tumor mouse model was supported by in vivo and ex vivo optical imaging, indicating a moderate CB_2_R specific binding. The fluorescent probe NIR760-XLP6 (**20**) was assessed for its potential as an imaging tool for pancreatic duct adenocarcinoma (PDAC). The evaluation showed that the probe achieved a good level of imaging contrast in both xenograft tumor and PDAC lymph node metastasis models. Additionally, the study confirmed the overexpression of CB_2_R in this kind of tumor. During the investigation, a substantial quantity of nonspecific binding of NIR760-XLP6 (**20**) was found. This is a typical difficulty in the creation of CB_2_R fluorescence probes [73].

The NIR760 fluorophore was used in conjunction with a selective CB_2_R quinolone structure at position 6 via a triazole-PEGylated linker to produce NIR760-Q (**22**) [74].

In this instance, the binding affinity and targeting specificity of CB_2_R were assessed in Jurkat cells, which inherently express CB_2_R. The NIR760-Q (**22**) compound has a high affinity in the nanomolar range, with K_d_ = 75.51 nM (Figure 7). Additionally, it demonstrates moderate specific binding, which aligns with the observed pattern seen in other charged NIR fluorescent dyes [74]. In comparison to the CB_2_R probe that was previously published, namely NIR760-mbc94 (**11**), NIR760-Q (**22**) exhibits improved absorption and emission properties while maintaining similar binding affinity and specificity. This study made the first use of optical imaging targeting CB_2_R in human tumor cells that endogenously express CB_2_R. The aggregated data suggest that NIR760-Q (**22**) exhibits promise as an imaging probe for the targeted imaging of CB_2_R, with potential applications in translational investigations.

In recent times, there has been an exploration of the potential attachment points for fluorescent dyes, specifically focusing on positions N1 and C3 of the oxoquinoline core [61]. Cooper et al. synthesized a collection of derivatives of 1,8-naphthyridin-2-(1H)-one-3-carboxamide, including the fluorophore BODIPY630/650-X at certain locations [75].

In comparison to previously known CB_2_R scaffolds, the naphthyridine moiety exhibits more hydrophilicity, which is expected to be advantageous in preventing undesired nonspecific binding. Regrettably, it has been observed that all fluorescent ligands connected to the N1 position have shown little affinity for CB_2_R. However, the derivative **21**, which is coupled to the C3 position and utilizes a glycine bridge, has demonstrated a significant affinity and selectivity for CB_2_R (pK_i_ = 6.33). This derivative functions as an inverse agonist [75].

Compound **21** has a noteworthy characteristic as it belongs to the category of infrequent CB_2_R ligands that demonstrate enhanced affinity following the incorporation of a fluorophore. Notwithstanding these favorable premises, it was shown that ligand **21** exhibited suboptimal imaging characteristics for CB_2_R in cellular contexts, which might be attributed to its pronounced membrane association and nonspecific intracellular accumulation [75].

In a recent structure–activity relationship (SAR) study focused on 4-oxo-1,4-dihydroquinoline analogs, a fluorescent CB_2_R ligand was successfully synthesized with attachment at the N1 position [70,76].

Spinelli et al., in a recent study, reported the synthesis of a comprehensive collection of CB_2_R fluorescent ligands. This was achieved by attaching 4-DMAP, NBD, and fluorescein cores to the N-adamantyl-4-oxo-1,4-dihydroquinoline-3-carboxamide pharmacophore. The lead drug, which contains a 4-DMAP fluorophore and a hexamethylene linker, had a favorable binding affinity towards the CB_2_R receptor (K_i_ = 130 nM) and exhibited a low level of radioligand displacement compared to the CB_1_R subtype (Figure 7). The researchers have introduced derivative **23** as an innovative candidate for the use of fluorescent-based competition binding tests and the identification of CB_2_Rs in certain cell types, as assessed using fluorescence-activated cell sorting (FACS) analysis. The obtained results from a preliminary competitive binding assay were used to measure the IC_50_ of two reference compounds, namely WIN55,212-2 and GW405833. Additionally, a saturation binding assay was conducted on CB_2_R-HEK293 cells, using GW405833 as the reference compound and [^3^H]-CP55,940 as the radioligand. Compound **7** was used as the fluo-ligand at a concentration of 100 nM with an incubation time of 90 min. The results obtained were found to be comparable to the Ki values reported in the existing literature [76].

Furthermore, experiments including saturation and binding tests were conducted in tumor cells that naturally express CB_2_Rs, providing further support for findings that have been previously documented for cells that have been genetically modified. Additional immunofluorescence tests were conducted using compound **23** at a concentration of 15 μM as a fluorescent probe for seeing CB_2_R in cells. These results demonstrated the significant specificity and reliability of compound **23** as a viable alternative to conventional radioligand assays.

### 4.4. Miscellaneous Fluorescent Probes

Since the presence of the original tetrahydrocannabinol (THC) core or analogs is often associated with a lack of CBR subtype selectivity and interference with other targets of the ECS, finding selective ligands harboring this moiety among all the CB_2_R functionalized probes is not frequent. Because of its unusual biological features and the fact that CBR subtype selectivity may be obtained via strategic substitutions, the tricyclic cannabinoid scaffold has recently received a great deal of interest. A series of fluorescent ligands was produced and physiologically tested, beginning with the bioisostere chromenopyrazole core [53].

**Figure 8:**
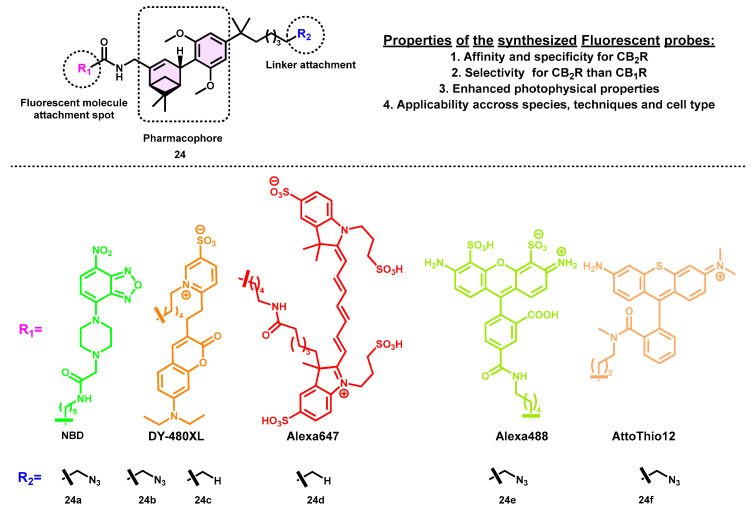
THC-based CB_2_ selective fluorescent probes and their properties.

Carreira and colleagues recently developed a series of fluorescent CB_2_R probes (**24a**–**f**) using NBD, DY-480XL, Alexa647, Alexa488, and AttoThio12 dyes (Figure 8, Table 1). The selection of fluorescent molecules was based on many criteria, including a significant Stokes shift, absorption and emission maxima that are red-shifted, a high molar extinction coefficient, appropriateness for Förster resonance energy transfer (FRET), and the possibility of use in ultra-high-resolution microscopy. Among all the synthetic molecules synthesized, a specific agonist, **24f,** has shown exceptional imaging characteristics and a remarkable binding affinity with the CB_2_ receptor with K_i_ = 4.7 nM and a notable selectivity (K_i_ CB_2_/CB_1_ ratio = 228). CB_2_R-selective fluorescent probes with unique photophysical characteristics were designed by attaching fluorescent dyes to amine building blocks through short linkers. The researchers used a previously reported high-recognition component that combines the characteristics of two CBR ligands, namely HU-308 and AM841. The docking simulations conducted on the DY-480-XL-carrying fluorescent azide derivative **24c** provided insights into the localization of the fluorescent tag in the extracellular region. Additionally, it was observed that the azide moiety forms a hydrogen bond with Leu191, which is likely a key factor contributing to the strong binding affinity exhibited by the two most effective azide derivatives as compared to their alkane counterparts. It is interesting to note that only changing the fluorescent dye moiety resulted in different efficacy (partial agonism for the molecule with AttoThio12 and agonism for the molecule attached with DY-480XL), all while maintaining extremely high functional selectivity over hCB_1_R. The efficacy of their probe showed it to be a valuable instrument in several experimental settings, including competition studies, flow cytometry, and the time-resolved confocal imaging of both human macrophages and mouse cells. In addition, the researchers assessed the kinetic binding characteristics using a newly developed TR-FRET-based assay, which is well suited to conducting high-throughput screening. Perhaps the most important point is that synthetic probes make it feasible to identify and monitor CB_2_R in cells that naturally express the receptor, which is not achievable with existing antibody technology [53].

Fluorescent imaging probes have been recognized as very sensitive technologies that provide a significant level of spatiotemporal resolution. In order to provide a reliable and verified translational pathway from preclinical pharmacological animal data to clinical applications, the Carreira research group endeavored to design a novel series of pyridine-based fluorescent probes for CB_2_R [54].

**Figure 9:**
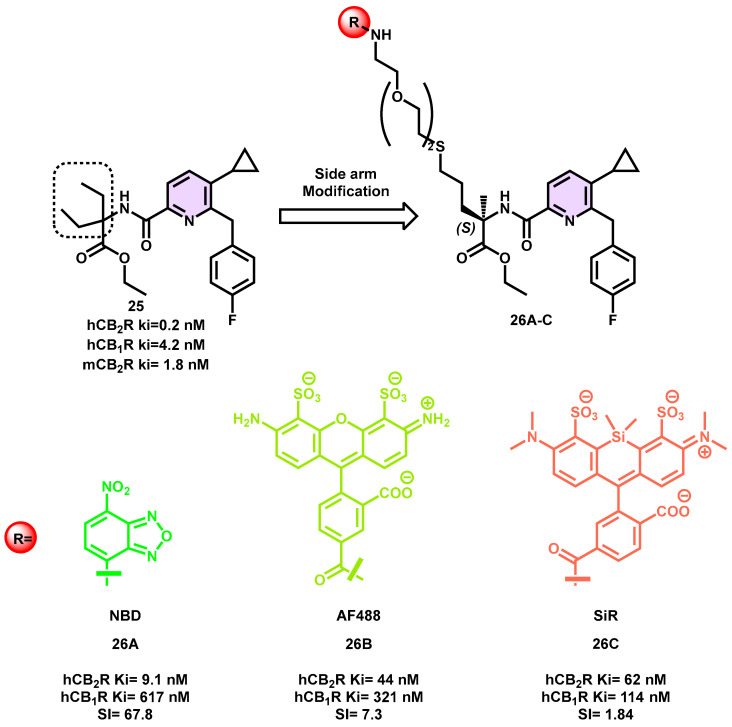
Pyridine-based CB_2_R fluorescent probe.

In a previous study, a set of fluorescent probes using a phytocannabinoid as the foundation was developed [53]. However, these probes exhibit suboptimal pharmacokinetic properties, limiting their potential for practical applications in translational research. Consequently, more progress was made using a reverse-design methodology, starting with a pharmacologically proven in vivo active compound class. This strategy aimed to meet many essential criteria, including affinity, potency, selectivity, chemical stability, water solubility, and membrane permeability. A ligand, denoted as **25**, has a preferential binding affinity in the picomolar range for both human and mouse CB_2_R receptors, and it demonstrates complete agonistic efficacy in the picomolar range (Figure 9) [77,78].

To facilitate the attachment of a fluorophore, a conjugation handle was necessary, based on the aforementioned lead. Initially, the use of docking research was employed to identify a potential linker and attachment site for the pharmacophore inside selective CB_2_R ligands based on pyrazine and pyridine [54].

A variety of fluorescent ligands were synthesized, whereby dyes were attached to the geminal diethyl group of the appropriately substituted pyridine moiety through a PEG linker. The process of optimizing the probes and selecting appropriate dyes was facilitated by conducting a thorough assessment of the absorption and emission spectra in a buffer solution. From the conducted investigations, it was discovered that all probes exhibited water solubility and did not demonstrate any inclination to form aggregates. A wide variety of lipophilicity values, ranging from 5.3 to 10.6, were detected for compound **26A**–**C** (Figure 9). This observation suggests that compound **26C**, which has an Alog *p* value of 10.6 and lacks negative charges according to its design, exhibits high permeability. These ligands were then subjected to in vitro biological evaluation. Among the derivatives examined, derivative **26A** emerged as the most effective ligand in terms of its potency, selectivity, and agonist functional features. However, due to the superior imaging characteristics of their respective dyes, only compounds **26B** and **26C** were subjected to further testing via fluorescence imaging. Overexpressing hCB_2_R-CHO cells and endogenously expressing native cells from human macrophages and mouse splenocytes were used with fluoroprobe **26C** for live-cell imaging of intracellular CB_2_R distribution using super-resolution confocal microscopy. During the FACS investigation, it was shown that both derivatives **26B** and **26C** exhibited a high degree of target specificity with little unspecific binding. Furthermore, probe **26C** was effectively used for the purpose of visualizing hCB_2_R in viable cells via the application of confocal imaging techniques. When injected intravenously, **26C** was shown to have a low toxicity profile in zebrafish larvae. It floated about the circulatory system for a while before it finally found its way to CB_2_R-expressing macrophages. This highlights the probe’s in vivo use and related issues, including biosafety, biodistribution, and cellular uptake. By providing a high-specificity, low-nanomolar-affinity probe for CB_2_R with full agonist efficacy, probe **26C** provides researchers with a novel and useful platform to study this receptor in a variety of relevant biological and pharmacological contexts [54].

**Figure 10:**
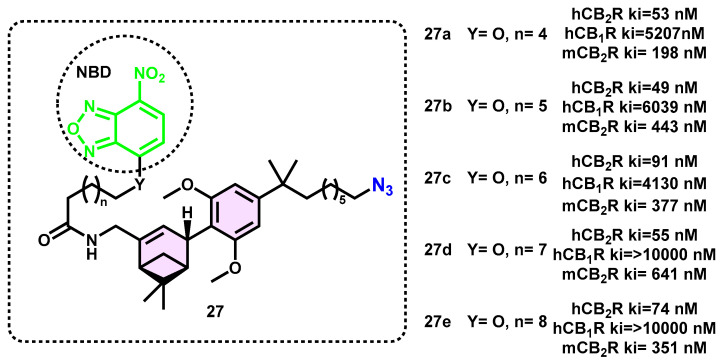
O-NBD and N-NBD probes for CB_2_R cell imaging.

Additional research and investigation into the development of a fluorescent probe that specifically targets CB_2_ receptors are needed. The Carriera group successfully pioneered the first ligand-directed covalent (LDC) labeling of CB_2_R using an innovative synthetic approach and the implementation of platform reagents. The affinity labeling technique, sometimes referred to as ligand-directed covalent (LDC) chemistry, enables the precise and selective fluorescent labeling of proteins that are naturally expressed within intricate and multifaceted systems. The LDC alteration facilitates the imaging and examination of CB_2_R while also preserving its capacity to bind various ligands at the orthosteric location. The use of in silico docking and molecular dynamics simulations is employed to provide guidance in the design of probes and evaluate the viability of tagging the CB_2_R with LDC. The study showcased the use of fluorogenic O-nitrobenzoxadiazole (O-NBD)-functionalized probes (**27a**–**e**) in a TR-FRET experiment to label the peripheral lysine residue of CB_2_R selectively and covalently (Figure 10). The confirmation of proof-of-concept was conducted rapidly using O-NBD probes, which subsequently led to the inclusion of advanced electrophiles that are appropriate for experimentation in living cells. In pursuit of this objective, innovative methodologies were devised to synthesize N-sulfonyl pyridone (NSP) and N-acyl-N-alkyl sulfonamide (NASA) LDC probes. These procedures facilitated the introduction of fluorophores via covalent bonding, making them ideal for cellular investigations. The LDC probes underwent characterization via the radioligand binding and TR-FRET assays.

The tendency of **27a**–**e** to bind more strongly to hCB_2_R than the mouse ortholog may be explained by the pattern of molecular interactions shown in MD simulations. This may be because of the amino acid substitutions Val261 ^6.51^ Ala, Ser90 ^2.60^ Asn, and Asn93 ^2.60^ Ile that occurred during the evolution from humans to mice. The mouse ortholog with an alanine residue lacks the Val261 ^6.51^ side chain’s hydrophobic interactions with the dimethyl group of the azidoheptyl substituent on the arene, which stabilize the molecule. Both the **27a** and **27c** probes produced weaker signals than **27b**. Similar to probe **27b**, both probes **27d** and **27e** were effective in covalently labeling their targets. Therefore, probes **27b**, **27d**, and **27e** were the most suited candidates for further research because of their high affinity and selectivity for CB_2_R in the radioligand assay and their robust signal in the TR-FRET test. The shorter, less lipophilic linker of **27b** was regarded as preferable to the linkers of **27d** and **27e** in this group due to its anticipated benefits in target selectivity and overall physicochemical qualities, most notably decreased lipophilicity.

Furthermore, the probes were used for the explicit purpose of visualizing CB_2_R in conventional and imaging flow cytometry, as well as in confocal fluorescence microscopy, using both overexpressing and endogenously expressing microglial live cells.

**Figure 11:**
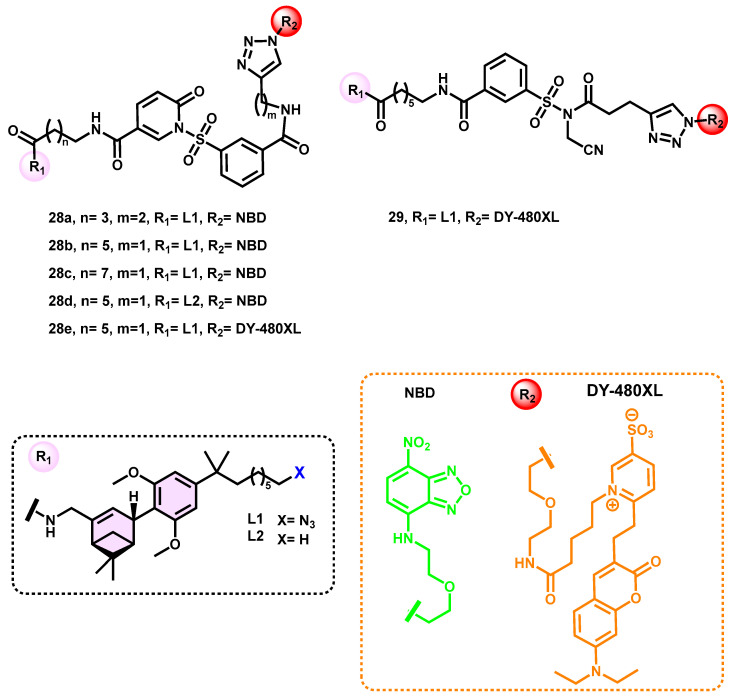
N-SP and NASA probes for CB_2_R live-cell imaging.

A significant improvement in labeling yields was seen when the incubation period of other sets of synthesized probes (**28a**–**e**) was prolonged to 20 h (Figure 11). Significantly, after a 20-h incubation period, **28e** exhibited a comprehensive covalent transfer of its fluorophore, as shown by the FRET experiment. Upon doing the experiment under conditions that mimicked physiological temperatures (namely, 37 °C), it was observed that the rate of cargo transfer was significantly increased. Namely, after a duration of 120 min, the cargo transfer reached a level of 53.5% ± 9.6%, indicating a substantial enhancement in the process. In the TR-FRET test, 119-fold selectivity for CB_2_R over CB_1_R was established via the measurement of saturation binding. The plasma membrane of the cells exhibited a strong DY-480XL signal, together with intracellular puncta that may indicate the presence of internalized receptors. The signal seen demonstrated colocalization between the SNAP-dye signal in the far-red range and the signal of interest. This colocalization provided confirmation that the tagging of SNAP tagged CB_2_Rs was effective using compound **29** [79].

## 5. Future Perspective and Critical Discussion

Small-molecule fluorescent ligands for use as tags for GPCRs have been in development throughout the last two decades. Innovation in chemical biology tools has been crucial to fostering this development. For instance, the last decades have seen significant progress in organic dye production, which is very helpful for the development of fluorescent ligands for GPCRs. Among the factors that drove the discipline out of its “comfort-zone” of radioligand substituents and into biochemical applications are the aforementioned advancements. This review delves into the use of fluorescence imaging techniques to study CB_2_ receptor expression, contributing to the advancement of our existing knowledge of CB_2_R and its therapeutic applications. Additionally, it sheds light on novel discoveries that have emerged from these investigations. The activation of CB_2_ has been shown extensively to suppress proliferation and promote autophagy and cell death, making it a promising target for therapy for cancer and other diseases [17,80]. According to the Drugs database of Global Data’s Pharma Intelligence Center, cannabinoid receptors have gained significant attention as a promising therapeutic target for the development of cannabis-based medications aimed at managing pain, neurological diseases, and inflammation. In the coming decade, one can expect a significant increase in the study of cannabinoid receptors to maximize their therapeutic potential. Cannabinoids based on CB_2_R have significant potential for the treatment of inflammation and pain management since they demonstrate quantifiable qualities of antipruritic, antinociceptive, and anti-inflammatory effects. It is anticipated that the systematic availability of more clinical data will facilitate the exploration and use of this intriguing collection of phyto- and synthetic cannabinoids. Many of the CB_2_ ligands suffer from poor pharmacokinetic properties, including high lipophilicity, low solubility, tight plasma protein binding, high in vivo clearance, and low oral bioavailability. In addition, residual CB_1_ agonism in subselective ligands further limits their application. Clearly, a balance must be established between excellent pharmacokinetic properties and high activity and selectivity for CB_2_R. An enhanced understanding of the kinetics involved in the binding of ligands to their respective targets, as well as the mechanisms behind biased signaling and allosterism in the CB_2_R receptor, together with the availability of more structural information pertaining to both antagonist- and agonist-bound CB_2_R, is expected to facilitate the development of more targeted approaches to drug design. The findings of this study have the potential to generate renewed optimism about the therapeutic capabilities of CB_2_R and contribute to a more comprehensive understanding of the endocannabinoid system (ECS). The application of AI and machine learning techniques may further enable the design and expeditious synthesis of smart ligands for bespoke applications in the immediate future.

## Figures and Tables

**Table 1:** Binding affinity (Ki) and selectivity values of the THC-based CB_2_ selective fluorescent probe.

		Ki (nM)			EC_50_ (nM)		
Compound	Dye	hCB_2_R	hCB_1_R	mCB_2_R	hCB_2_R	hCB_1_R	mCB_2_R
**24a**	NBD	4.2	>10,000	n.d.	n.d.	>10,000	n.d.
**24b**	DY-480XL	99	4031	1986	>10,000	>10,000	>10,000
**24c**	DY-480XL	21	2378	1459	171	2152	118
**24d**	Alexa647	2565	>10,000	>10,000	25	2565	370
**24e**	Alexa488	268	>10,000	1204	n.d	n.d.	n.d.
**24f**	Atto Thio12	4.7	1075	1.1	5.6	>10,000	17

## Data Availability

Data sharing is not applicable.

## Abbreviations

GPCRs = G protein-coupled receptors; ECS = endocannabinoid system; CB_1_R = Cannabinoid type 1 receptor; CB_2_R = Cannabinoid type 2 receptor; hCB_1_R = Human Cannabinoid type 1 receptor; hCB_2_R = Human Cannabinoid type 2 receptor; THC = tetrahydrocannabinol; AEA = Arachidonoyl Ethanolamide/Anandamide; CNS = central nervous system; FRET = Förster resonance energy transfer; TR-FRET = time-resolved Förster resonance energy transfer; PET = positron emission tomography; BRET = bioluminescence resonance energy transfer; NK cells = natural killer cells; NBD = nitrobenzoxadiazole; CHO-K1 cells = Chinese hamster ovary cells; cAMP = Adenosine-3′, 5′-cyclic monophosphate, or cyclic AMP; SAR = structure-activity relationship; PDAC = pancreatic duct adenocarcinoma; DMAP = 4-Dimethylaminopyridine; FACS = fluorescence-activated cell sorting; PEG = Polyethylene glycol; LDC= ligand-directed covalent; NIRF = near-infrared fluorophores; QDs = quantum dots; DBT = delayed brain tumor; SI = Selectivity index; NASA = N-acyl-N-alkyl sulfonamide; NSP = N-sulfonyl pyridone.
